# Factors associated with dental anxiety in patients treated at an integrated dental clinic: a cross-sectional study

**DOI:** 10.3389/froh.2025.1689805

**Published:** 2025-09-25

**Authors:** Luis Chauca-Bajaña, Alba Pérez-Jardón, Carlos Carpio-Cevallos, Andrea Ordoñez Balladares, Patricio Proaño-Yela, Byron Velásquez Ron, Leonardo Javier Siguencia Suarez, Carla Verenice Romo Olvera, Diana Orellana Guerrero, Mario Pérez-Sayáns

**Affiliations:** 1 College of Dentistry, University of Guayaquil, Guayas, Ecuador; 2 Oral Medicine, Oral Surgery and Implantology Unit (MedOralRes), Faculty of Medicine and Dentistry, Universidade de Santiago de Compostela, Santiago de Compostela, Spain; 3 ORALRES GROUP, Health Research Institute of Santiago de Compostela (IDIS), Santiago de Compostela, A Coruna, Spain; 4 College Dentistry, University Bolivariana del Ecuador, Durán, Ecuador; 5 Carrera de Odontología, Facultad de Odontología, Universidad de Las Américas (UDLA), Quito, Ecuador; 6 Department Prosthesis Research, Universidad de las Américas (UDLA), Quito, Ecuador; 7 Instituto de los Materiales de Santiago de Compostela (iMATUS), Santiago de Compostela, A Coruna, Spain

**Keywords:** dental anxiety, anxiety factors, prevalence, oral health, psychological factors

## Abstract

**Background:**

Dental anxiety is a widespread issue that significantly impacts oral health behaviors, often leading individuals to postpone or avoid dental care. This avoidance can result in more complex and invasive treatments. This study aimed to evaluate the levels of dental anxiety and associated factors among patients attending the dental clinic at the University of Guayaquil, Ecuador.

**Materials and methods:**

A total of 389 patients were assessed using three validated instruments: the Modified Dental Anxiety Scale (MDAS), the Dental Anxiety Short Scale (SDAI), and the Dental Anxiety and Fear Diagnosis (DAYMO). Statistical analyses included bivariate tests and correlation analyses using Spearman's rho and Pearson's chi-square tests.

**Results:**

Among the participants, 63.8% were female and 36.3% were male. Women reported significantly higher levels of anxiety across all assessment tools (*p* < 0.05). Based on the SDAI, 47% of patients exhibited moderate to severe anxiety, whereas 19% reported high anxiety levels on the MDAS. The DAYMO instrument revealed that 51.2% of participants experienced mild anxiety, and 27.8% had moderate anxiety. Common anxiety triggers included fear of pain, past negative dental experiences, and concerns about needles or oral hygiene practices.

**Conclusions:**

Dental anxiety was prevalent among patients, particularly among women, with most experiencing mild to moderate levels. These findings underscore the importance of addressing psychological factors in dental care settings to improve patient experience and outcomes.

## 1 Introduction

Dental anxiety affects many people and is perceived as extreme insecurity related to visits to the dentist or dental treatments (1). Dental anxiety can be caused by fear of pain, previous negative experiences, the feeling of lack of control or simply fear of the unknown (2, 3). This anxiety can trigger a response in the autonomic nervous system, thus leading to increases in involuntary bodily functions, such as blood pressure, heart rate, respiratory rate, gastrointestinal motility and cardiac output (4). The stress that arises in response to dental treatment can be triggered by a variety of factors, ranging from a clearly identifiable cause to more vague stimuli, or it may even occur without any apparent reason (5). Dental anxiety is a complex response that activates multiple systems within the body in reaction to a perceived threat or danger, significantly impacting daily life and acting as a barrier to seeking and receiving dental care (6). This anxiety manifests in three main spheres: the cognitive, characterized by negative thoughts and worries; the psychophysiological, involving physical responses such as increased heart rate; and the behavioral, marked by avoidance of dental appointments (7). Recent evidence indicates that dental anxiety is not only related to previous negative experiences or fear of pain but also to a combination of cognitive and non-cognitive factors. Cognitive aspects, such as catastrophic thinking, negative beliefs, and perceived lack of control, have been shown to significantly intensify anticipatory fear before treatment (8, 9). In contrast, non-cognitive factors, including traumatic dental events, family influence, cultural background, and personality traits such as neuroticism or introversion, also play a relevant role in shaping the patient's anxiety response (10, 11). The interplay of these factors highlights the multidimensional nature of dental anxiety and underlines the importance of considering both psychological and contextual determinants when assessing and managing this condition.

Dental phobia, on the other hand, represents a more intense form of anxiety, characterized by persistent concern regarding specific objects or situations, such as injections or dental procedures in general, which can trigger panic episodes accompanied by sweating and trembling (12). A meta-analysis showed that the prevalence of dental anxiety in Chinese adults was 35.4% (13). A systematic review and meta-analysis reported that the estimated overall prevalence of high and severe dental fear and anxiety (DFA) in adults was 15.3%, 12.4%, and 3.3%, respectively (14). People with severe dental anxiety tend to avoid dental appointments, thereby compromising their oral health, increasing the risk of dental pain, and leading to more invasive treatments (15). A study by Thomson et al. in 2009 (16) revealed that DFA usually occurs in childhood, adolescence or even later in life; hence, it is important to use a reliable and valid assessment tool to properly diagnose dental anxiety before a patient visits a dentist (17). There are several scales designed to measure dental anxiety, among the most commonly used being the DASS-21, which was not included in this study as it also assesses depression (18–21). Among these, the Modified Dental Anxiety Scale (MDAS) is one of the most widely used questionnaires to assess dental anxiety in patients seeking dental care, regardless of the type of procedure they are facing (16, 22). In 1969, Norman Corah (15) created the Dental Anxiety Scale (DAS) to measure pre-procedure anxiety in patients; subsequently, in 1995, this questionnaire evolved into the Modified Dental Anxiety Scale (MDAS), leading to improvements in patient care and experience in the dental environment (23). In 1995, Stouthard, Groen and Mellenbergh developed the nine-item Simplified Dental Anxiety Inventory (SDAI); the SDAI was created based on an analysis of the Dental Anxiety Inventory and has been proven to be a reliable and valid tool (24). Additionally, the SDAI has been shown to be strongly correlated with other dental anxiety scales, such as the Dental Anxiety Scale (DAS), thereby indicating its efficacy for assessing anxiety in patients before they undergo dental procedures (25).

The SDAI, MDAS and DAYMO assess dental anxiety from distinct yet complementary perspectives. MDAS focuses on anticipatory anxiety in specific dental treatment scenarios, while the SDAI evaluates a broader range of emotional and behavioral responses to dental procedures. Additionally, the Dental Anxiety and Your Memory of the Dentist (DAYMO) scale, used as a complementary measure, identifies specific triggers of dental anxiety, such as fear of pain or needles.

This study aimed to better understand dental anxiety and its contributing factors among patients at a comprehensive dental clinic in Guayaquil, Ecuador. To do so, we considered it essential to assess dental anxiety from different complementary perspectives provided by the three selected questionnaires (MDAS, SDAI, and DAYMO) each of which offers a slightly different focus.

The null hypothesis was that there are no significant differences in dental anxiety levels among the different questionnaires used nor in relation to associated factors, such as gender and anxiety-triggering factors, among patients attending the comprehensive dental clinic in Guayaquil, Ecuador.

## 2 Materials and methods

### 2.1 Study design and study setting

This cross-sectional observational study was conducted among patients who received care at the comprehensive dental clinic of the Faculty of Dentistry at the University of Guayaquil. Specifically, participants were treated by students in the eighth, ninth, and tenth semesters during academic Cycle II of the 2024–2025 period. The STROBE reporting guidelines were followed.

#### 2.1.1 Inclusion criteria

- Patients aged ≥18 years who attended the comprehensive dental clinic of the Faculty of Dentistry, University of Guayaquil, during academic Cycle II (2024–2025).
- Patients who provided written informed consent and voluntarily agreed to participate.
- Patients who fully completed the three questionnaires (MDAS, SDAI, and DAYMO).

#### 2.1.2 Exclusion criteria

- Patients with cognitive impairments or psychiatric diagnoses that could interfere with the reliable completion of the questionnaires.
- Patients undergoing emergency dental treatment (e.g., acute pain, infection, trauma), as their clinical condition could bias the assessment of dental anxiety.
- Patients who declined participation or submitted incomplete questionnaires.

### 2.2 Ethical consideration and informed consent

Ethical approval was obtained from both the Dean of the Faculty and the Bioethics Committee of the USC (Reference: USC01/2024), in accordance with the ethical principles outlined in the Declaration of Helsinki. All participants were informed of the study objectives and procedures and provided written informed consent prior to their inclusion in the study.

### 2.3 Sample size and sampling technique

Two statistical approaches were considered for the sample size estimation. First, based on previously published data regarding differences in mean anxiety levels measured using the Modified Dental Anxiety Scale (MDAS), an *a priori* power analysis was performed. To detect a mean difference of 0.8 with a standard deviation of 1.5 between men and women, assuming equal variances, a 95% confidence level, and a power of 90%, a minimum of 75 individuals per group was required. This calculation was conducted using Epidat 4.2, resulting in a total required sample size of 150 participants.

Additionally, for the correlational analysis, a proper priori power calculation was performed using G Power 3.1 software. Considering an expected correlation coefficient of *r* = 0.192, a two-tailed test, an alpha level of 0.05, and a desired power of 0.95, the minimum required sample size was estimated to be 389 participants. The actual sample included in the study matched this requirement, ensuring adequate statistical power for the analyses conducted.

### 2.4 Research instrument

The participants answered three questionnaires anonymously in the waiting room of the comprehensive clinic. The first questionnaire was the Modified Dental Anxiety Scale (MDAS), which was validated in Spanish by Coolidge et al. (26). In addition, this scale has also been validated in several languages (27–31). The MDAS consists of five items that assess anticipatory anxiety in common dental situations, including waiting for a check-up, sitting in the dentist's chair, anticipating drilling, undergoing scaling, and receiving an anesthetic injection. Each item is rated on a 5-point scale (1 = not anxious, 2 = slightly anxious, 3 = fairly anxious, 4 = very anxious, 5 = extremely anxious). The scores are summed to obtain a global index of dental anxiety, widely validated as a reliable screening tool in different languages and populations. Total scores <9 indicate mild or no anxiety, 9–12 indicate moderate anxiety, 13–14 indicate high anxiety, and ≥15 suggest severe anxiety or dental phobia (16).

The SDAI was originally developed in English by Irene Aartman in 1998 (32) and we have used a version translated into Spanish. The SDAI comprises nine items designed to evaluate both emotional and behavioral reactions to dental stimuli, including nervousness before treatment, avoidance behaviors, and physiological symptoms (e.g., sweating, trembling). Each item is scored on a 5-point Likert scale (33). Total scores range from 9 to 10 (minimal anxiety), 11 to 19 (mild anxiety in specific situations), 20 to 27 (moderate anxiety with some self-control), and 28 to 36 (severe dental anxiety) (34, 35).

The DAYMO questionnaire was developed in Spanish by the authors and previously tested in a pilot study, showing good internal consistency (Cronbach's alpha = 0.817). It consists of items addressing specific anxiety-triggering factors such as fear of pain, needles, noises, or the dentist's judgment of oral hygiene. Each item is scored on a 5-point Likert scale (33), with total scores categorized as follows: < 9 = mild or no anxiety, 9–12 = moderate anxiety, 13–15 = high anxiety, and >15 = severe anxiety. Unlike the MDAS and SDAI, which primarily measure general anticipatory or situational anxiety, the DAYMO provides a more detailed assessment of contextual triggers relevant to dental settings.

The following independent variables were studied: Gender, Marital Status, Socioeconomic status, Level of Education, Number of Children. Socioeconomic status and level of education were determined based on participants' self-reported categorization. As dependent variables, the results of the SDAI, MDAS, and DAYMO Tests.

### 2.5 Statistical analysis

A multivariate analysis was conducted to evaluate the association between qualitative variables from questionnaires and dental fear and anxiety. Relative frequencies and measures of central tendency (mean, median, standard deviation and variance) were calculated. Spearman's correlation tests were used to assess the relationship between demographic characteristics and dental anxiety. The normality of data distributions was verified using the Kolmogorov–Smirnov test. The associations between sociodemographic variables and the classification of fear and anxiety were determined using Pearson's chi-square test. Bivariate analysis was applied to evaluate the simultaneous effect of the associated factors that would explain the fear and anxiety scores by means of multiple linear regression analysis after verifying that the data met the necessary assumptions (i.e., randomness of the dependent variable and linearity). Results were presented through tables and graphs using SPSS Statistical Software version 27 (36). In addition, the Cronbach alpha coefficient of each scale was calculated, and sociodemographic data such as sex, marital status, socioeconomic level and level of education were also recorded. In all analyses, a *p*-value less than 0.05 was considered statistically significant.

## 3 Results

A total of 389 patients were included in this study, of which 63.8% were women and 36.3% were men. A descriptive analysis of the sociodemographic data of the sample is shown in Table 1.

**Table 1:** Descriptive data.

Socio-demographic variables		*n*	%
Gender	Female	248	63.8
	Male	141	36.2
Marital status	Single	178	45.8
	In a relationship	50	12.9
	Living together	43	11.1
	Divorced	16	4.1
	Married	102	26.2
Education level	Completed primary	39	10.0
	Incompleted primary	8	2.1
	Secondary education	153	39.3
	Incompleted secondary	34	8.7
	Completed profession	71	18.3
	Incompleted profession	79	20.3
	Postgraduated	5	1.3
Socioeconomic status	Low	84	21.6
	Medium	294	75.6
	High	11	2.8

The anxiety-inducing factors related to dental visits are presented in Table 2, which shows the questions for the different tests and the participants' responses.

**Table 2:** Questionnaires from the different tests analyzed.

Test SDAI		*n*	%
Do I start to get nervous when the dentist invites me to sit in the chair?	Never	151	38.8%
	Rarely	82	21.1%
	Sometimes	84	21.6%
	Always	41	10.5%
	Very frequent	31	8.0%
When I know that the dentist is going to extract a tooth, I feel really scared in the waiting room?	Never	98	25.2%
	Rarely	102	26.2%
	Sometimes	90	23.1%
	Always	59	15.2%
	Very frequent	40	10.3%
When I'm on my way to the dentist's office and I think about the sound of the bur, does it make me want to turn away and not go?	Never	164	42.2%
	Rarely	81	20.8%
	Sometimes	67	17.2%
	Always	42	10.8%
	Very frequent	35	9.0%
Do I want to leave the dentist's office when I think the dentist won't explain what they're going to do to my teeth?	Never	150	38.6%
	Rarely	84	21.6%
	Sometimes	70	18.0%
	Always	48	12.3%
	Very frequent	37	9.5%
At the moment when the dentist prepares the syringe with the anesthesia injection, do I tightly close my eyes?	Never	97	24.9%
	Rarely	88	22.6%
	Sometimes	68	17.5%
	Always	94	24.2%
	Very frequent	42	10.8%
Do I sweat and tremble in the waiting room when I think it's my turn to go into the consultation?	Never	199	51.2%
	Rarely	83	21.3%
	Sometimes	45	11.6%
	Always	26	6.7%
	Very frequent	36	9.3%
When I go to the dentist's office, do I get anxious just thinking about whether he or she will have to use the burr on me?	Never	162	41.6%
	Rarely	88	22.6%
	Sometimes	59	15.2%
	Always	36	9.3%
	Very frequent	44	11.3%
When I am sitting in a treatment chair and I don't know what the dentist is doing in my mouth, do I get nervous and sweat?	Never	142	36.5%
	Rarely	112	28.8%
	Sometimes	64	16.5%
	Always	36	9.3%
	Very frequent	35	9.0%
¿On my way to the dentist's office, does the thought of sitting in the treatment chair make me nervous?	Never	188	48.3%
	Rarely	84	21.6%
	Sometimes	56	14.4%
	Always	26	6.7%
	Very frequent	35	9.0%
Test MDAS		*n*	%
If you had to go to the dentist tomorrow for a check-up, how would you feel about it?	Relaxed, not anxious	209	53.7%
	slightly anxious	122	31.4%
	Fairly anxious	39	10.0%
	Very anxious and restless	15	3.9%
	Extremely anxious (sweaty, tachycardic, with a feeling of severe illness)	4	1.0%
¿When you are waiting your turn at the dentist's office in the chair, how do you feel?	Relaxed, not anxious	200	51.4%
	slightly anxious	128	32.9%
	Fairly anxious	41	10.5%
	Very anxious and restless	16	4.1%
	Extremely anxious (sweaty, tachycardic, with a feeling of severe illness)	4	1.0%
When you are in the dentist's chair waiting while the dentist prepares the drill to begin work on your teeth, ¿how do you feel?	Relaxed, not anxious	148	38.0%
	slightly anxious	157	40.4%
	Fairly anxious	55	14.1%
	Very anxious and restless	20	5.1%
	Extremely anxious (sweaty, tachycardic, with a feeling of severe illness)	9	2.3%
Imagine that you are in the dentist's chair for a dental cleaning. While you wait the dentist or hygienist takes out the instruments that will be used to scrape your teeth around the gums. ¿How do you feel?	Relaxed, not anxious	170	43.7%
	slightly anxious	140	36.0%
	Fairly anxious	61	15.7%
	Very anxious and restless	10	2.6%
	Extremely anxious (sweaty, tachycardic, with a feeling of severe illness)	8	2.1%
If you are going to be injected with a needle with local anesthetic for your dental treatment, how do you feel?	Relaxed, not anxious	122	31.4%
	slightly anxious	146	37.5%
	Fairly anxious	80	20.6%
	Very anxious and restless	29	7.5%
	Extremely anxious (sweaty, tachycardic, with a feeling of severe illness)	12	3.1%
Test DAYMO		*n*	%
Do you experience fear of dental pain?	Never	63	16.2%
	Rarely	121	31.1%
	Sometimes	90	23.1%
	Always	66	17.0%
	Very frequent	49	12.6%
Have you had negative dental experiences?	Never	147	37.8%
	Rarely	117	30.1%
	Sometimes	79	20.3%
	Always	16	4.1%
	Very frequent	30	7.7%
Do you have fear of needles or injections?	Never	108	27.8%
	Rarely	116	29.8%
	Sometimes	69	17.7%
	Always	58	14.9%
	Very frequent	38	9.8%
Do you have Claustrophobia or fear of closed spaces?	Never	227	58.4%
	Rarely	70	18.0%
	Sometimes	45	11.6%
	Always	20	5.1%
	Very frequent	27	6.9%
Do you often worry about the dentist's judgment about your oral hygiene or the condition of your teeth?	Never	86	22.1%
	Rarely	82	21.1%
	Sometimes	96	24.7%
	Always	71	18.3%
	Very frequent	54	13.9%
Do you experience fear of noises and smells associated with the dentist?"	Never	171	44.0%
	Rarely	93	23.9%
	Sometimes	70	18.0%
	Always	26	6.7%
	Very frequent	29	7.5%
Do you have fear or anxiety towards invasive or unknown dental procedures?	Never	90	23.1%
	Rarely	114	29.3%
	Sometimes	84	21.6%
	Always	47	12.1%
	Very frequent	54	13.9%

Association analyses using the Chi-square test between socio-demographic variables and anxiety assessments (SDAI, MDAS, and DAYMO) revealed significant differences related to gender, with *p*-values of 0.025 for SDAI, 0.01 for MDAS, and <0.001 for DAYMO. However, no statistically significant associations were observed for other variables, such as marital status, education level, socioeconomic status, and number of children (Table 3).

**Table 3:** Analysis of sociodemographic variables in relation to the SDAI, MDAS, and DAYMO tests.

Variables		Test SDAI		Test MDAS		Test DAYMO	
		*χ* ^2^	*p*	χ^2^	*p*	χ^2^	*p*
Sociodemographic	Gender	11.171	0.025	11.337	0.01	20.118	<0.001
	Marital status	18.385	0.302	16.250	0.180	21.735	0.152
	Educational level	25.157	0.397	24.551	0.138	28.829	0.227
	Socioeconomic status	14.881	0.061	4.018	0.674	9.619	0.293
	Number of children	21.317	0.264	14.363	0.705	18.152	0.446

Both the SDAI and MDAS demonstrated high reliability, with Cronbach's alpha coefficients of 0.905 and 0.875, respectively. The DAYMO showed good but slightly lower reliability, with Cronbach's alpha coefficient of 0.817. 47% of patients who completed the SDAI reported moderate or severe levels of anxiety during their visit to the dentist. However, MDAS results indicated that 54% of patients experimented with mild or no anxiety, while 19% reported high or severe anxiety. The DAYMO findings revealed that 51.2% of patients experienced mild anxiety and 27.8% experienced moderate anxiety during dental consultations. Only 5.1% of patients reported severe anxiety. A deeper analysis of the DAYMO results shows that men were more likely to report mild anxiety (58.2%) compared to women (47.2%), while women were more likely to report moderate anxiety (35.1%) compared to men (14.9%). Regardless of the assessment tool (SDAI, MDAS or DAYMO), women exhibited higher anxiety levels than males (Table 4).

**Table 4:** Anxiety rating according to each scale.

Patient gender	Total *n* = 389					***p* Value (χ²)**
	Female *n* = 248	%	Male *n* = 141	%	Total *n* (%)	
**TEST MDAS**						
Mild or no anxiety	129	52	84	59.6	213 (54.8)	0.01
Moderate anxiety	58	23	42	29.8	100 (25.7)	
High anxiety	28	11.3	7	5	35 (9.0)	
Severe anxiety/phobia	33	13.3	8	5.7	41 (10.0)	
**TEST SDAI**						
No anxiety	13	5.2	14	9.9	27 (6.9)	0.025
Minimal anxiety	6	2.4	10	7.1	16 (4.1)	
Mild anxiety	107	43.1	54	38.3	161 (41.4)	
Moderate anxiety	56	22.6	37	26.2	93 (23.9)	
Severe anxiety	66	26.6	26	18.4	92 (23.7)	
**TEST DAYMO**						
No anxiety	21	8.5	22	15.6	43 (11.1)	0.001
Minimal anxiety	12	4.8	7	5	19 (4.9)	
Mild anxiety	117					

MDAS, modified dental anxiety scale; SDAI, simplified dental anxiety index; DAYMO, diagnosis of dental anxiety and fear.

**Figure 1:**
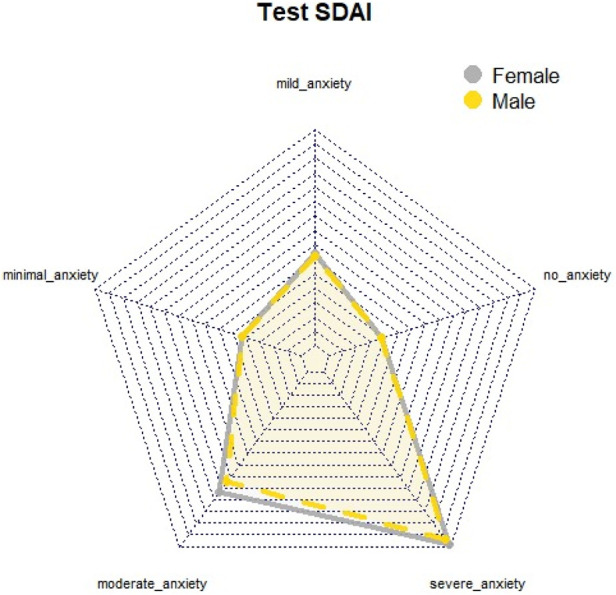
Radial graph SDAI test.

**Figure 2:**
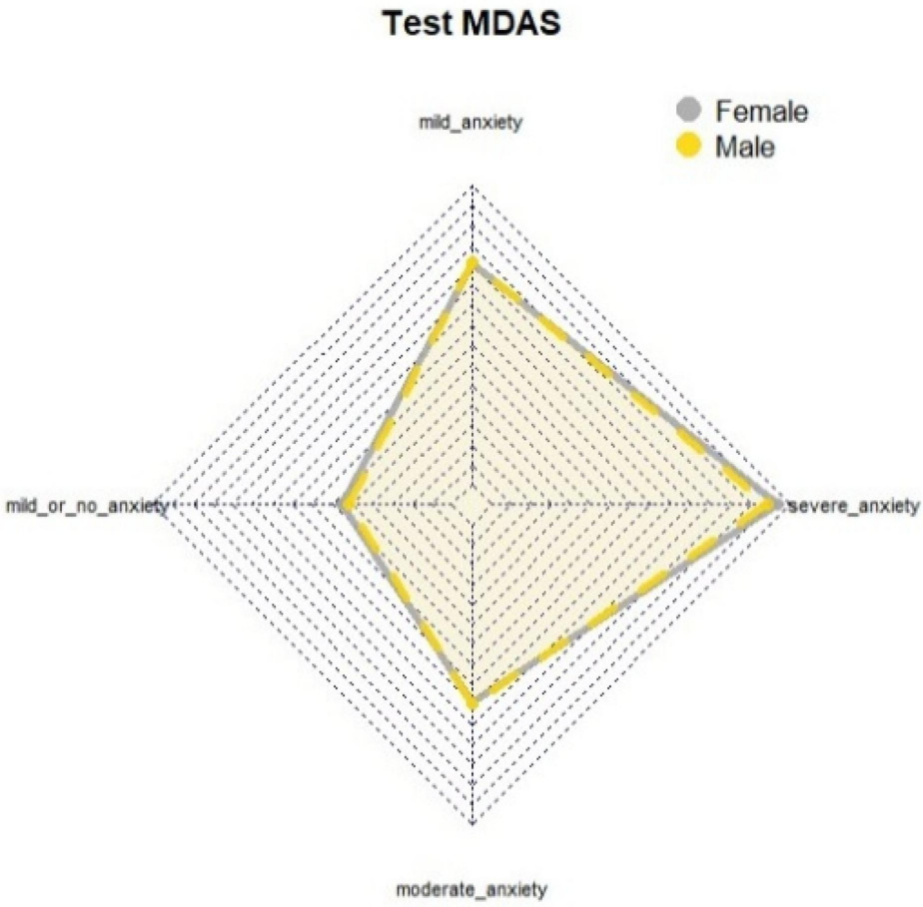
Radial graph MDAS test.

**Figure 3:**
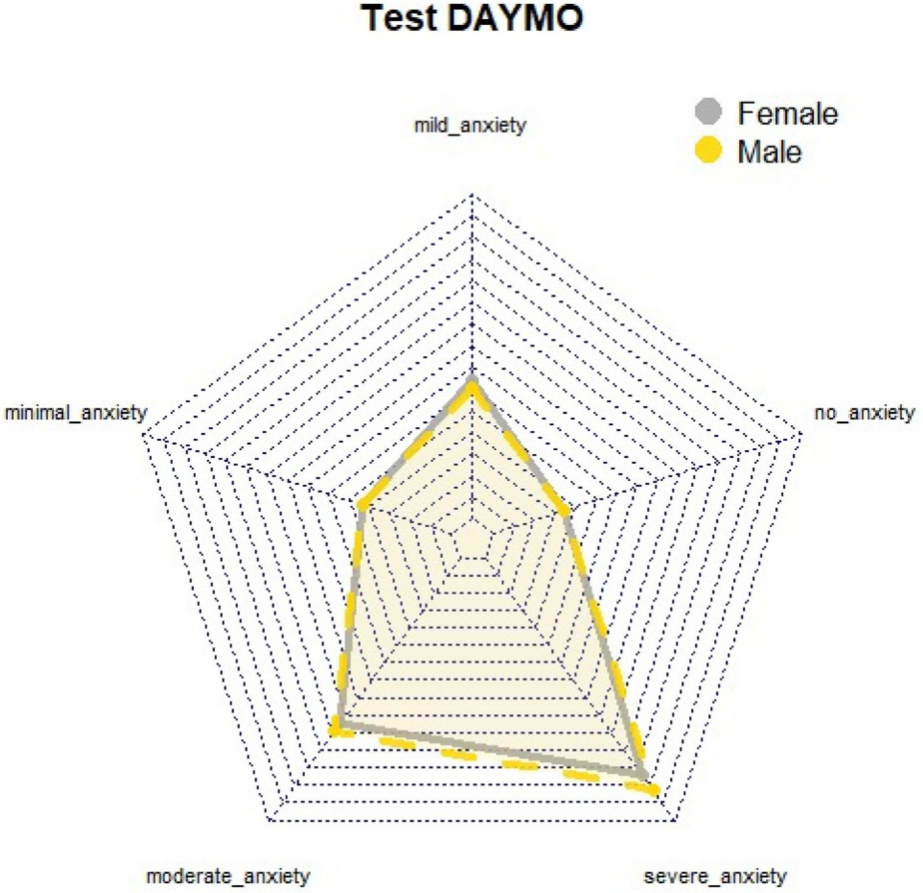
Radial graph DAYMO test.

The results indicate that women exhibit higher levels of dental anxiety compared to men, with a predominance of both mild and severe anxiety. Additionally, while both men and women reported anxiety levels approaching the threshold of severe anxiety on the SDAI, this pattern was not observed on the MDAS or DAYMO (Figure 1). Graphical analyses of perimeter distances between axes in relation to MDAS anxiety levels suggested that both genders reported anxiety levels nearing severe or phobic thresholds (Figure 2). This finding was further supported by the DAYMO results, which showed that both men and women reported anxiety levels very close to severe anxiety (Figure 3).

The variety of anxiety-triggering factors during dental visits was remarkable. Fear of treatment pain, previous negative dental experiences, and apprehension about needles or injections emerged as prominent concerns. In the SDAI test, 11% of patients felt high anxiety when thinking about the use of the dental drill and the preparation of the syringe with anesthesia. In the MDAS test, 53% felt relaxed during dental check-ups and 51% in the waiting room, although 40% experienced mild anxiety in the dentist's chair and 37% when anticipating an injection. According to the DAYMO test, 31% had occasional fear of dental pain and 29% felt some apprehension about needles, but the majority did not have negative dental experiences or fear of sounds or odors from the dental office.

All three tests (SDAI, MDAs, and DAYMO) show positive and significant correlations with each other, suggesting that they are measuring similar aspects of anxiety. The correlations are statistically significant at a confidence level of 0.01, indicating that it is highly unlikely these correlations are due to chance.

Statistically significant correlations were observed between the total scores of the SDAI, MDAS and DAYMO. A strong positive association between these scores was observed, supported by strong and consistently high correlations (Table 5).

**Table 5:** Pearson correlation between tests.

Correlations	Total score SDAI test	Total score MDAS test	Total score DAYMO test
Total score SDAI test			
Pearson correlation	1	.698**	.741**
Covariance	80.663	24.11	40.897
Total score MDAS test			
Pearson correlation	.698**	1	.615**
Covariance	24.11	14.788	14.54
Total score DAYMO test			
Pearson correlation	.741**	.615**	1
Covariance	40.897	14.54	37.8

Values represent Pearson correlation coefficients between the tests.

**Indicates a correlation significant at the 0.01 level (*p* < 0.01).

By using binary linear regression, the analysis examines whether gender significantly predicts anxiety scores across these different assessment tools. The results in Table 6 further clarify these associations. While the MDAS shows a statistically significant difference between genders (*p* = 0.02), indicating that gender has a meaningful impact on anxiety levels measured by this scale, the associations for SDAI (*p* = 0.13) and DAYMO (*p* = 0.07) did not reach statistical significance. The regression coefficients (B values) indicate the direction of these relationships, with negative values suggesting that females (coded as the reference group) exhibited higher anxiety scores than males (Table 6).

**Table 6:** Binary linear regression of tests vs. gender.

Variable (Test vs. Gender)	R	Constant	B	F	*p*-value	CI 95%
SDAI vs. Gender	0.126	23.955	−2.346	6.218	0.13	21.282–26.628
MDAS vs. Gender	0.156	10.969	−1.244	9.609	0.02	9.830–12.109
DAYMO vs. Gender	0.137	19.199	−1.756	7.451	0.07	17.372–21.026

R, correlation coefficient; B, regression coefficient; F, F statistic.

**Figure 4:**
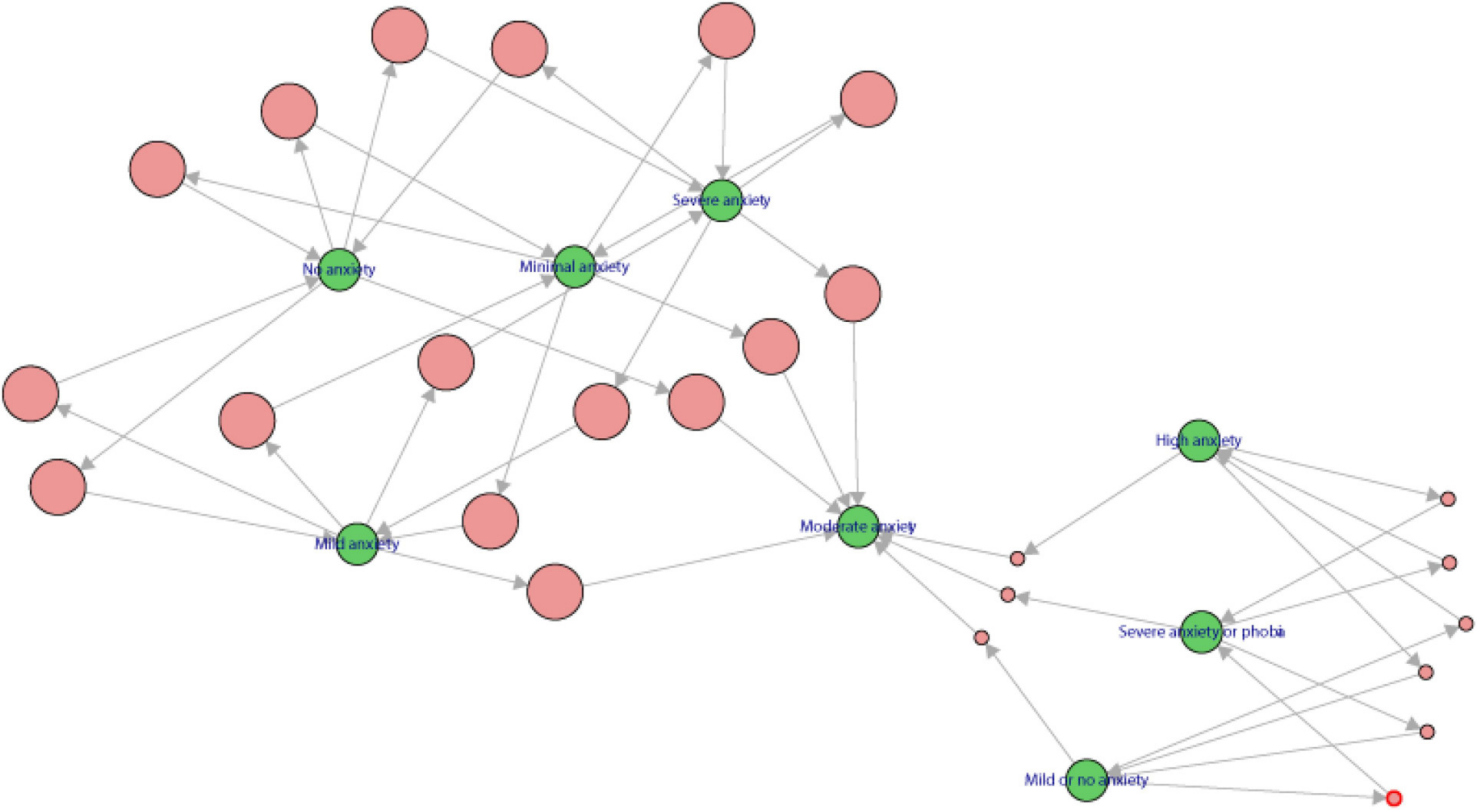
Network of interactions between anxiety levels and associated factors.

In addition, a network analysis was performed showing how different levels of anxiety are related, from “No anxiety” and “Mild anxiety” to more intense states such as “Severe anxiety” and “Phobia”. The green nodes represent these levels, while the red ones indicate associated factors that influence them. The arrows suggest how some levels can lead to more severe ones. This approach allows a better understanding of the connections and pathways that can cause anxiety to increase, providing a clearer perspective on how these states develop and worsen (Figure 4).

## 4 Discussion

Dental anxiety, characterized by apprehension, nervousness, or fear related to dental care, remains a prevalent concern in dental practice (37). This study aimed to evaluate anxiety levels and associated factors among patients treated at a comprehensive dental clinic using 3 questionnaires, i.e., the MDAS, SDAI and DAYMO. Our findings revealed significant levels of anxiety among participants, with 19% experiencing high or moderate anxiety according to MDAS, 47% reporting moderate anxiety according to SDAI, and 33% exhibiting moderate to severe anxiety according to DAYMO. These results align with previous research, such as Gil-Abando et al. (38), who studied 200 patients and found that 10% had moderate anxiety and 3.5% had severe anxiety according to the MDAS Scale. Similarly, Metin-Gürsoy et al. (39), reported severe anxiety in 34.1% and moderate anxiety in 27.6% of participants. These findings highlight the constant presence of moderate to severe anxiety, thus emphasizing the importance of addressing this concern in dental care to improve patient experiences. Sivaramakrishnan et al. (40) concluded that 23.7% of the participants experienced moderate anxiety, 11.4% felt very anxious, and 4.6% suffered from dental phobia. In contrast, White et al. (41) found that 19% of the total study population exhibited moderate to high dental anxiety, while 6.8% had high levels of dental anxiety. Muneer MU et al. (37) determined that 75% reported having dental anxiety. Other studies, such as those by Alemany-Martínez et al. (42) and Caltabiano et al. (43), showed that women had higher levels of dental anxiety. Saatchi et al. (44) found that women exhibited significantly higher levels of anxiety (*p* < 0.001) and fear (*p* = 0.003) compared to men in a study of 473 patients. Similarly, Erten et al. (45) reported that women tended to experience greater anxiety, with a mean score of 9.5 ± 4.1 vs. 7.9 ± 3.3 in men. Furthermore, do Nascimento et al. (46) concluded that anxiety was more prevalent in women, (20.7% vs. 11.3%) in men (*p* = 0.995). These findings indicate a consistent trend in the literature, highlighting that women tend to experience higher levels of anxiety in dental contexts.

This study highlights a notable disparity in the levels of high and severe anxiety, evidencing a significant prevalence in women in contrast to men. While educational attainment could play an important role, as shown in other studies (47–49), our current study did not find a significant association between educational level and anxiety related to dental care. Other scales, such as the SDAI, were used. Liu Y et al. (50), found that individuals with periodontal disease had an average dental anxiety score of 23.4 ± 8.5, while those without the disease had a score of 22.6 ± 8.6. On the other hand, in the study by Murillo-Benítez et al. (51), the mean SDAI anxiety score was 27.2 ± 12.5, highlighting that 70% of the women presented moderate or high levels of anxiety. In Nepal, research at the BP Koirala Institute of Health Sciences found that 2% of patients exhibited extreme anxiety (MDAS ≥ 19), 20.67% experienced high anxiety, and 51.33% had moderate anxiety, indicating that more than half of the patients experienced some degree of anxiety (52). Similarly, in Lebanon, a study conducted in 29 private dental clinics revealed that 31.5% of patients suffered from dental anxiety, while 22.4% had dental phobia, suggesting that fear of dental procedures is a common concern across different populations (53). In Saudi Arabia, a study at Al-Jouf University found that 51.6% of patients experienced dental anxiety, with 22.1% at a moderate level, 17.1% at a high level, and 12.4% at an extreme level (54). In line with these findings, another study conducted at the Faculty of Dentistry Hospital in Jeddah reported that 48.3% of patients experienced dental anxiety (score >15), while 2.5% had dental phobia (score >16), further confirming the significance of this issue in the region (55).

The results of the research highlight that 47.6% of people had moderate to severe anxiety, with a higher prevalence of anxiety in women. This finding highlights a statistically relevant connection between the levels of anxiety assessed through the SDAI and the gender characteristics of the patients. In addition, the relationships between the questionnaires provide valuable insights into anxiety assessment. The association between the SDAI score and MDAS score suggested a connection between mild and severe anxiety levels. However, when contrasting the SDAI and DAYMO, discrepancies are observed in the identification of mild anxiety. Despite this, the high concordance between MDAS and DAYMO in the absence of anxiety, as well as in the detection of moderate and high levels, highlights the consistency in those specific ranges. These findings underscore the complexity of measuring and understanding anxiety in the dental context, highlighting the need to consider multiple assessment tools to capture diverse aspects of patients' experience of anxiety.

The clinical relevance of this study lies in its ability to provide dental professionals with valuable information regarding the prevalence and associated factors of dental anxiety. Identifying that a significant proportion of patients experience moderate to severe levels of anxiety, along with triggering factors such as fear of pain and negative past experiences, enables dentists to adjust their treatment approaches. Furthermore, the observed gender differences in anxiety levels underscore the need to implement personalized strategies that enhance the patient experience, promoting greater adherence to preventive care and reducing treatment avoidance.

Furthermore, our findings are consistent with international evidence on dental anxiety. In a Chinese implant surgery context, moderate and high preoperative dental anxiety were found in 66.6% and 11.9%, respectively (56). In the Netherlands, a representative adult sample showed dental fear in 24.3% of respondents, with dental phobia present in 3.7%, and women reporting more severe fear (57). These comparisons confirm that dental anxiety is a widespread phenomenon, often more pronounced among women and younger populations. This underscores the importance of broad strategies—like clear communication, pain control, and relaxation techniques—while adapting them to sociocultural contexts.

## 5 Strengths and limitations

This study has several strengths. First, it included a relatively large sample size (*n* = 389), which provided adequate statistical power for the analyses and enhanced the reliability of the findings. Second, we employed three validated and complementary instruments (MDAS, SDAI, and DAYMO), allowing for a more comprehensive assessment of dental anxiety than studies relying on a single measure. Third, to our knowledge, this is one of the few investigations exploring dental anxiety in an Ecuadorian population, thereby contributing novel data from a Latin American context where evidence is still limited. Finally, the rigorous application of statistical analyses, including correlation and regression, ensured a robust evaluation of associations between anxiety and demographic variables.

Nevertheless, some limitations should be acknowledged. The cross-sectional design prevents establishing causal relationships between anxiety and associated factors. The use of self-reported questionnaires may introduce response or recall bias, as participants might underreport or overreport their true levels of anxiety. In addition, because the sample was recruited from a single university dental clinic, the findings may not be fully generalizable to other populations or clinical settings. Lastly, the simultaneous use of different anxiety scales, while enriching the analysis, may lead to variability in the reported prevalence across instruments, which should be interpreted with caution.

Despite these limitations, the results highlight the importance of understanding and addressing dental anxiety in daily practice. For many patients, fear of pain, previous negative experiences, and needle apprehension can turn a simple dental visit into a highly stressful experience. In response, dentists can make a meaningful difference by implementing effective strategies, such as explaining each procedure clearly, using distraction techniques such as music or virtual reality, and ensuring a more relaxed atmosphere in the office. Moreover, the fact that women presented higher levels of anxiety reinforces the need for an empathetic and personalized approach, providing a space where patients feel heard and understood. Incorporating tools such as the MDAS or SDAI in the initial assessment would allow early identification of individuals who require special anxiety management, thereby optimizing the patient experience and promoting better adherence to dental treatments. In the long term, this approach not only improves the relationship between dental professionals and patients but also contributes to better oral health outcomes in the population. Finally, our findings emphasize the importance of continuing education in patient psychology for dental professionals to improve treatment adherence and reduce avoidance of dental visits due to fear or anxiety.

Future research should include longitudinal and multicenter studies to evaluate changes in dental anxiety over time, as well as randomized controlled trials to assess the effectiveness of targeted interventions. These approaches would provide more precise and evidence-based guidelines for dental professionals in managing dental anxiety and enhancing the quality of patient care.

## 6 Conclusions

### 6.1 Prevalence of anxiety

A significant proportion of patients experienced varying levels of dental anxiety.

Gender Differences: Women exhibited higher levels of moderate and severe anxiety compared to men, according to all anxiety assessment tools (MDAS, SDAI, DAYMO).

### 6.2 Reliability of assessment tools

The MDAS and SDAI demonstrated high reliability (Cronbach's alpha coefficients of 0.905 and 0.875, respectively), while the DAYMO showed slightly lower reliability (Cronbach's alpha of 0.817).

### 6.3 Factors influencing anxiety

Fear of pain, previous negative dental experiences, and fear of needles were identified as the primary triggers of dental anxiety.

### 6.4 Correlation Among scales

A strong correlation was observed between the total scores of the SDAI, MDAS, and DAYMO scales, indicating consistency in measuring anxiety across different tools.

## Data Availability

The original contributions presented in the study are included in the article/Supplementary Material, further inquiries can be directed to the corresponding author.
